# Effects of Photodynamic Therapy and Glucocorticosteroids on Salivary Oxidative Stress in Oral Lichen Planus: A Randomized Clinical Trial

**DOI:** 10.3390/antiox14081017

**Published:** 2025-08-20

**Authors:** Patryk Wiśniewski, Magdalena Sulewska, Jagoda Tomaszuk, Anna Zalewska, Sara Zięba, Aleksandra Pietruska, Emilia Szymańska, Katarzyna Winnicka, Mateusz Maciejczyk, Małgorzata Żendzian-Piotrowska, Małgorzata Pietruska

**Affiliations:** 1 Department of Periodontal and Oral Mucosa Diseases, Medical University of Bialystok, ul. Waszyngtona 13, 15-269 Bialystok, Poland; magdalena.sulewska@umb.edu.pl (M.S.); jagoda.tomaszuk@umb.edu.pl (J.T.); malgorzata.pietruska@umb.edu.pl (M.P.); 2 Department of Restorative Dentistry, Medical University of Bialystok, ul/Marii Skłodowskiej Curie 24a, 15-089 Białystok, Poland; anna.zalewska1@umb.edu.pl (A.Z.); sara.zieba@umb.edu.pl (S.Z.); 3 Student’s Research Group at the Department of Periodontal and Oral Mucosa Diseases, Medical University of Bialystok, ul. Waszyngtona 13, 15-269 Bialystok, Poland; perio@umb.edu.pl; 4 Department of Pharmaceutical Technology, Medical University of Białystok, Mickiewicza 2c, 15-222 Białystok, Poland; emilia.szymanska@umb.edu.pl (E.S.); katarzyna.winnicka@umb.edu.pl (K.W.); 5 Department of Hygiene, Epidemiology and Ergonomics, Medical University of Bialystok, Mickiewicza 2c, 15-222 Białystok, Poland; mat.maciejczyk@gmail.com (M.M.); mzpiotrowska@gmail.com (M.Ż.-P.)

**Keywords:** oral lichen planus, photodynamic therapy, topical corticosteroids, salivary biomarkers, oxidative stress, antioxidant enzymes

## Abstract

**Objective:** This study aimed to assess the impact of photodynamic therapy (PDT) and topical glucocorticosteroids (GKS) on total oxidant status (TOS), total antioxidant capacity (TAC), and oxidative stress index (OSI) in the saliva of patients with oral lichen planus (OLP). **Methods:** Ninety patients with histopathologically confirmed OLP were randomly assigned to either the PDT group (*n* = 50) or the GKS group (*n* = 40). Unstimulated saliva samples were collected before treatment and at 1, 3, and 6 months post-therapy. TOS, TAC, and OSI were determined using colorimetric assays. **Results:** Both PDT and GKS significantly reduced TOS over the entire observation period. TAC decreased persistently after GKS but remained stable after PDT except for an initial decline. OSI was significantly lower immediately after PDT but did not show sustained differences. Overall, PDT more effectively and durably restored redox balance compared to GKS. **Conclusions:** Photodynamic therapy demonstrates superior long-term efficacy in modulating oxidative stress markers in saliva, supporting its role as a promising alternative to topical corticosteroids in managing OLP. Clinically, these findings suggest that PDT may offer a non-invasive, recurrence-reducing, and steroid-sparing treatment alternative for OLP, potentially improving long-term patient outcomes and reducing side effects associated with prolonged corticosteroid use.

## 1. Introduction

Oral lichen planus (OLP) is a chronic, autoimmune disease of the oral mucosa classified among oral potentially malignant disorders (OPMDs) [1]. It is estimated to affect approximately 1% of the general population, occurring more frequently in women over the age of forty [2,3]. Clinically, it presents in various forms, ranging from asymptomatic white striations to painful erosions and ulcers, which significantly impair patients’ quality of life [4]. Of particular concern is the fact that OLP lesions may undergo malignant transformation into oral squamous cell carcinoma (OSCC) [1].

**Figure 1:**
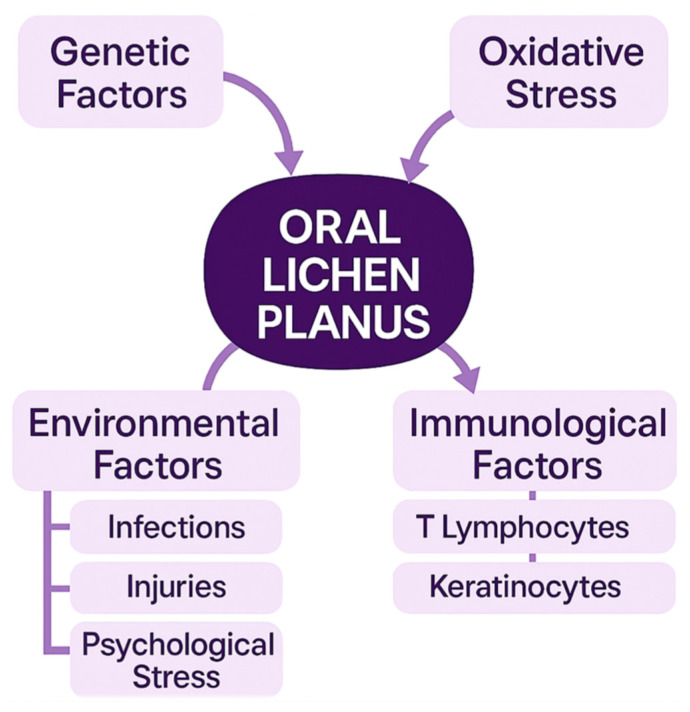
Etiological factors of oral lichen planus.

The etiology of OLP has not yet been fully elucidated. Disturbances in immune response, especially the cytotoxic activation of T lymphocytes, are considered crucial, leading to keratinocyte apoptosis and damage to the epithelial basement membrane [5,6]. The development of the disease is also influenced by genetic, environmental, and infectious factors, including hepatitis C virus infection, chronic stress, tobacco use, and exposure to certain medications and heavy metals [7,8,9,10,11,12]. Increasing attention has been paid to the role of oxidative stress (OS) in the pathogenesis of OLP. Excessive production of reactive oxygen species (ROS) initiates lipid peroxidation processes, damages keratinocyte proteins and DNA, and amplifies the inflammatory response, creating a vicious cycle that perpetuates chronic inflammation [13] (Figure 1).

Studies on the redox profile of saliva have demonstrated that patients with OLP exhibit increased concentrations of oxidative stress markers, such as malondialdehyde (MDA) and 8-hydroxy-2′-deoxyguanosine (8-OHdG), as well as reduced activity of key enzymatic and non-enzymatic antioxidants [14]. For example, Darczuk et al. (2016) reported elevated thiobarbituric acid reactive substances (TBARS), reduced glutathione (GSH), and total antioxidant capacity (TAC) in unstimulated saliva, while Rezazadeh et al. (2023) found significantly lower GSH, catalase (CAT), and free thiol levels compared to healthy controls [15,16]. A recent meta-analysis (2021) confirmed a significant redox imbalance in both saliva and serum of OLP patients, characterized by elevated MDA, nitric oxide (NO), and 8-OHdG levels, and reduced TAC and uric acid (UA) activity [14]. To comprehensively assess the redox balance, cumulative indices are employed: total oxidant status (TOS), total antioxidant capacity (TAC), and the oxidative stress index (OSI), the values of which may reflect the intensity of oxidative processes accompanying the disease [17,18].

To date, the treatment of OLP has primarily relied on symptomatic therapy. Topically administered glucocorticosteroids (GKS) remain the standard of care, effectively reducing the severity of inflammation and alleviating pain [19]. However, chronic use of steroids carries the risk of numerous adverse effects, including mucosal atrophy, fungal infections, and, less commonly, systemic disturbances such as adrenal insufficiency [20,21]. In recent years, photodynamic therapy (PDT) has attracted growing interest, as it offers selective cytotoxic effects on pathological cells and a favorable safety profile, representing a promising alternative to corticosteroids [22]. The introduction of PDT into dental practice is hindered by the lack of photosensitizers specifically formulated for the oral mucosa—topical dermatological preparations show poor adhesion and limited effectiveness [23]. In this study, a proprietary PDT protocol using a 5-aminolevulinic acid-based emulgel with high mucosal adhesion was applied [24,25].

It is noteworthy that the currently available therapeutic modalities predominantly target clinical symptoms without directly addressing pathogenetic processes such as chronic oxidative stress. Identifying a therapeutic strategy that not only reduces inflammation but also modulates the impaired redox balance could significantly enhance treatment effectiveness and reduce the risk of relapse and disease progression [14].

The aim of this study was to evaluate the impact of PDT and topical GKS therapy on total oxidant status, total antioxidant capacity, and the oxidative stress index in the saliva of patients with OLP.

## 2. Materials and Methods

### 2.1. Study Participants

This investigation was designed as a single-center, prospective, randomized clinical trial conducted at the Department of Periodontal and Oral Mucosal Diseases, Medical University of Bialystok, between September 2021 and January 2023. The study protocol received approval from the Bioethics Committee of the Medical University of Bialystok (decision number: APK.002.372.2021). All participants were informed about the objectives and procedures of the study and provided written informed consent. The study was designed, conducted, and reported in accordance with the Consolidated Standards of Reporting Trials (CONSORT 2010) [26].

A total of 100 individuals with clinically and histopathologically confirmed OLP were enrolled. After applying exclusion criteria, data from 90 patients (72 women and 18 men) aged between 29 and 88 years (mean age: 60 ± 11.7 years), in whom 161 disease foci were identified in total, were subjected to statistical analysis.

Inclusion criteria were age over 18 years and a histopathological diagnosis of OLP. The diagnostic criteria for OLP are not precisely defined and have evolved over recent years. According to expert consensus, the diagnosis should be based on a number of clinical and histopathological features. Clinical criteria include (1) bilateral, symmetrical whitish lesions affecting the buccal mucosa and/or tongue and/or lips and/or gingiva; (2) whitish papular lesions and slightly elevated lines over the mucosa, with or without erosions; and (3) occasionally, desquamative gingivitis as an additional manifestation. Histopathological criteria comprise (1) a well-defined lymphocytic infiltrate confined to the superficial lamina propria; (2) vacuolar degeneration of the basal cell layer and/or suprabasal layers with keratinocyte apoptosis; and (3) epithelial thinning and ulceration with a mixed inflammatory infiltrate in the atrophic form [27]. Patients with the reticular or erosive form of OLP were eligible for inclusion. None of the lesions showed features of epithelial dysplasia. No patient was taking medications or dietary supplements with antioxidant properties (e.g., vitamin C, vitamin E), and none were active smokers. Exclusion criteria included pregnancy, breastfeeding, severe systemic diseases (including oncological, dermatological, and hepatic disorders), known photosensitivity or allergy to the photosensitizer, prior OLP treatment within the previous 6 months, use of immunosuppressive or immunomodulatory drugs, psychiatric disorders, and the presence of other pathological lesions in the oral cavity.

### 2.2. Study Groups

**Figure 2:**
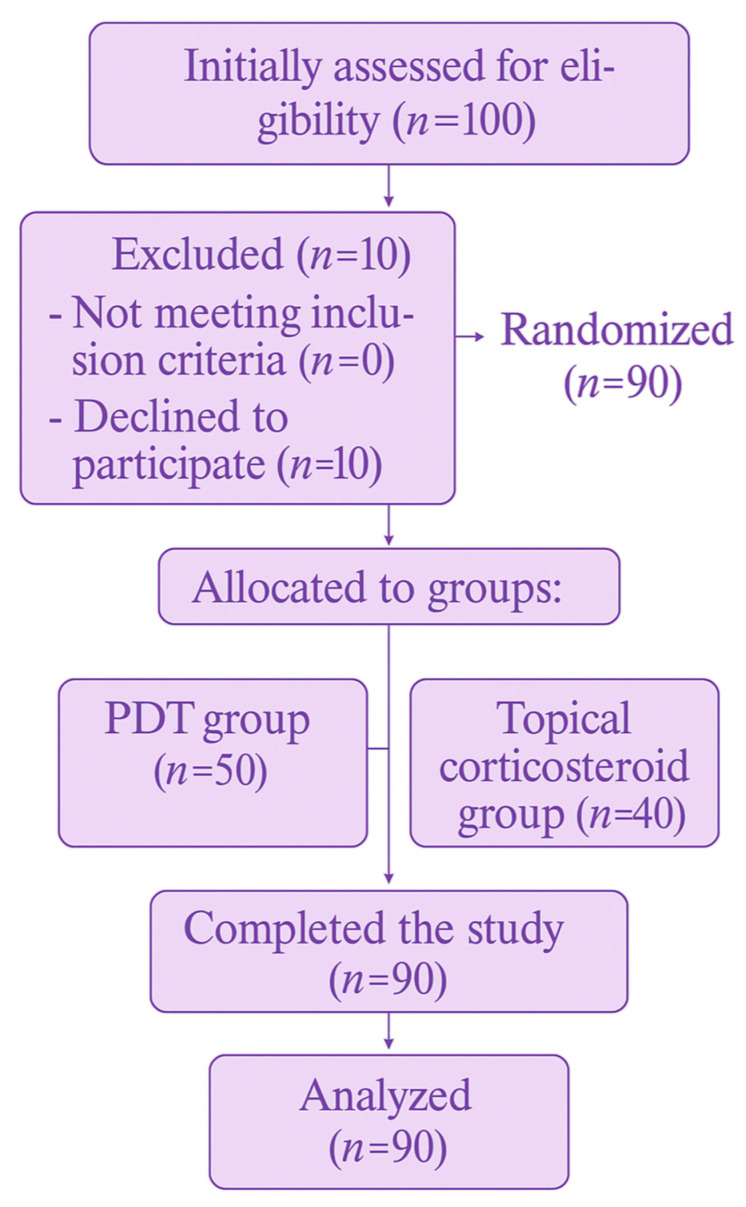
Patient flow chart.

Participants were randomly allocated into two treatment groups using block randomization, according to a pre-generated randomization list created in Microsoft Excel (Figure 2). Randomization was performed by a single investigator. The study followed a single-blind design, in which blinding applied only to the clinical examiner. The examiner was unaware of the type of procedure performed and was not allowed to ask participants any questions regarding the treatment. Similarly, patients were instructed not to discuss the nature or progress of their therapy with the examiner. Complete blinding of participants was not feasible due to the inherently different nature of the two therapeutic procedures being compared.

The PDT group received a novel mucoadhesive composition in the form of an emulgel containing 5% (*w*/*w*) 5-aminolevulinic acid (5-ALA) (patent P.443813) according to the proprietary protocol (ALA-PDT). In brief, after drying the oral mucosa, the 5-ALA preparation was applied to the gently dried lesion in a layer approximately 2 mm thick, twice: 40 and 20 min before the planned illumination. The treated area was covered with gauze and secured using sterile compresses to prevent saliva from reaching the site. Illumination was performed using an LED light source (FotoSan^®^ 630, CMS Dental, Copenhagen, Denmark) emitting light at a wavelength of 630 nm, with a power output of 300 mW and an energy density of 108 J/cm^2^. The beam was delivered in non-contact mode at approximately 2 mm from the lesion, applied in a single continuous stage without interruptions for 6 min per square centimeter of the lesion. The full treatment protocol consisted of five sessions, conducted once a week.

The second group was treated with a topical corticosteroid—clobetasol propionate (Clobederm 0.5 mg/g), applied twice daily for 14 days.

### 2.3. Saliva Collection

Unstimulated saliva samples were collected at four time points: prior to treatment initiation and at 1, 3, and 6 months after completion of therapy. Strict pre-collection recommendations were provided: patients refrained from food and beverage intake (except water) for at least two hours, from using oral hygiene products, and from taking any medications for at least eight hours before sampling.

Collection was performed in the morning hours (between 8:00 and 10:00 a.m.) in a dedicated room, with patients seated and leaning their heads forward after a 5 min adaptation period. The oral cavity was rinsed with distilled water, and the first minute of saliva flow was discarded. Subsequent portions were collected by spitting over a 15 min period until a total volume of 5 mL was obtained. Samples were placed in Falcon tubes, stored on ice, centrifuged (4 °C, 3000× *g*, 20 min), and frozen at −80 °C until further analysis [28].

### 2.4. Biomarker Assays

In the saliva samples, the total antioxidant capacity and total oxidant status were determined. All measurements were performed using colorimetric methods. Additionally, the oxidative stress index was calculated.

TOS was assessed using the method of Erel [29], which is based on the oxidation of Fe^2+^ to Fe^3+^ in the presence of oxidants in the sample, with measurement of absorbance changes of xylenol orange. Results were expressed in nmol H_2_O_2_ equiv/min/mg protein.

TAC was measured according to Erel [30], based on the ability of the samples to neutralize the ABTS*^+^ radical cation. Results were standardized to protein content and expressed in μmol Trolox/mg protein.

Total protein content was determined using the bicinchoninic acid assay (Thermo Scientific PIERCE BCA Protein Assay kit, Rockford, IL, USA).

The OSI was calculated as the ratio of TOS to TAC (OSI = TOS/TAC × 100%).

### 2.5. Statistical Analysis

The required sample size was estimated using the G*Power 3.1 software (Universität Düsseldorf, Düsseldorf, Germany), based on pilot data from a preliminary study on oxidative stress markers in patients with oral lichen planus treated with photodynamic therapy and corticosteroids. To detect a medium effect size (f = 0.25) with a statistical power of 0.80 and a significance level of α = 0.05 in repeated measures ANOVA within–between interaction (two groups × four time points), the minimum sample size was calculated to be 86 participants. To account for potential dropouts, a final target sample size of 100 individuals was established. Statistical analysis of the obtained data was performed using GraphPad Prism 10.5 (GraphPad Software, La Jolla, CA, USA). The Shapiro–Wilk test was applied to assess data normality, while Levene’s test was used to verify homogeneity of variance. The analyses demonstrated that the data did not meet the criteria for normal distribution; therefore, non-parametric tests were applied for further calculations. Friedman repeated measures analysis of variance by ranks was performed, and Dunn’s test was used for post hoc comparisons. A correction for multiple comparisons was applied. Statistical significance was set at *p* < 0.05.

## 3. Results

A total of 90 patients with OLP were enrolled in the study. In the group treated with photodynamic therapy, 50 participants completed the analysis, whereas in the group treated with topical corticosteroid therapy, 40 participants completed the study (Table 1).

The assessment focused on changes in the levels of oxidative stress markers in unstimulated saliva: total oxidant status, total antioxidant capacity, and oxidative stress index, measured at four time points: before treatment, immediately after therapy, and at 3 and 6 months following the completion of treatment.

### 3.1. Effect of PDT on TOS, TAC, and OSI Values in Saliva of Patients with OLP

**Figure 3:**
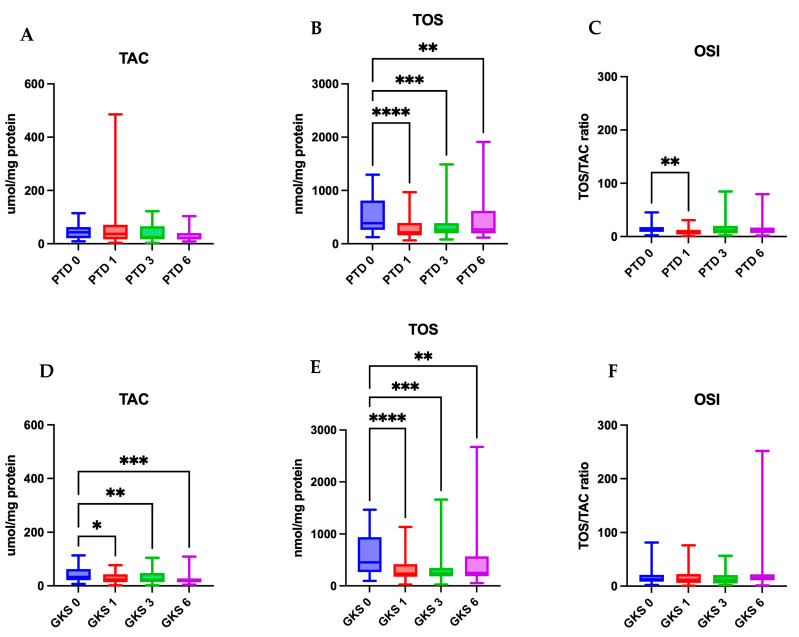
Comparison of salivary total antioxidant capacity, total oxidant status, and oxidative stress index measured at four time points in OLP patients treated with PDT (**A**–**C**) or GKS (**D**–**F**). TAC—total antioxidant capacity; TOS—total oxidant status; OSI—oxidative stress index. PDT0, GKS0—before treatment; PDT1, GKS1—immediately after treatment; PDT3, GKS3—after 3 months; PDT6, GKS6—after 6 months. * *p* < 0.05, ** *p* < 0.01, *** *p* < 0.001, **** *p* < 0.0001. [TAC] = umol/mg protein, [TOS] = nmol/mg protein, [OSI] = 1.

The mean TOS values in the PDT group before treatment were 520.2 ± 357.8 nmol/mg protein and decreased significantly immediately after therapy (294.7 ± 199.7 nmol/mg; *p* < 0.0001). During subsequent follow-up, a gradual increase in TOS was observed; however, the values remained significantly lower compared to baseline (*p* < 0.01). Total antioxidant capacity did not change significantly over time (*p* > 0.05), although at the third month after treatment, a downward trend was noted (*p* < 0.1). The mean OSI prior to therapy was 14.15 ± 9.20 and decreased significantly to 9.58 ± 7.45 immediately after treatment (*p* < 0.01). At 6 months after completion of therapy, OSI values increased again, reaching approximately 19, although this change did not attain statistical significance compared to baseline (Table 2, Figure 3).

### 3.2. Effect of GKS on TOS, TAC, and OSI Values in Saliva of Patients with OLP

In the GKS group, the mean pre-treatment TOS was 572.6 ± 380.5 nmol/mg protein, which decreased significantly after therapy (331.8 ± 263.8 nmol/mg; *p* < 0.0001). This effect persisted during subsequent follow-up visits (*p* < 0.01). The TAC before treatment was 43.92 ± 28.19 µmol/mg protein and significantly declined after therapy and throughout the observation period (*p* < 0.05). The OSI decreased slightly after therapy (mean from 18.51 to 16.24); however, these changes did not reach statistical significance (*p* > 0.05) (Table 2, Figure 3).

### 3.3. Comparison of the Effect of PDT and GKS on Selected Oxidative Stress Parameters in Saliva of Patients with OLP

No significant differences in TOS medians between the groups were observed at any time point during follow-up (*p* > 0.05). Comparison of TAC showed that immediately after treatment, values were significantly higher in the PDT group compared to the GKS group (*p* < 0.05). At six months, a trend towards higher TAC values in the PDT group was noted (*p* < 0.1). The oxidative stress index differed significantly between groups immediately after therapy, being lower in the PDT group (*p* < 0.05). In subsequent time points, the differences were not statistically significant (Table 3).

## 4. Discussion

Oral lichen planus is a chronic inflammatory disease of the oral mucosa whose management remains a significant therapeutic challenge. The variability and severity of clinical symptoms considerably reduce patients’ quality of life [4]. Current treatment protocols focus primarily on symptom control and are largely palliative in nature [31]. Therefore, there is an ongoing search for therapeutic modalities that could target causative mechanisms, including oxidative stress.

Topical administration of GKS remains the gold standard in the treatment of OLP [19]. GKS drugs exhibit potent anti-inflammatory effects, effectively alleviating pain and improving patients’ comfort [32]. However, GKS therapy carries the risk of adverse effects such as mucosal thinning, xerostomia, stomatodynia, or local hypersensitivity. As a result, there are limitations regarding the frequency and duration of their use [20,21].

Photodynamic therapy has emerged as an alternative treatment for OLP and has gained increasing popularity in recent years. Although numerous studies have demonstrated clinical efficacy comparable to that of corticosteroids, standardized therapeutic protocols specifying the type of photosensitizer, irradiation parameters, and treatment schedules are still lacking [33]. It is important to emphasize that the outcomes achieved using the proprietary photodynamic therapy protocol appear promising [24,25].

In the present study, we demonstrated that both GKS and PDT influence oxidative stress parameters in the unstimulated saliva of patients with OLP.

A statistically significant reduction in TOS values was observed following both photodynamic therapy PDT and GCS treatment. Tunelli-Akbay et al. (2017) reported elevated salivary TOS levels and OSI in patients with OLP [34]. OSI reflects the predominance of oxidative processes over antioxidant capacity, a phenomenon that undoubtedly occurs in the etiopathogenesis of OLP [35]. In our study, OSI decreased significantly immediately after PDT. However, in the longer follow-up, its value did not differ significantly from baseline. No significant changes in OSI were observed after GCS therapy. To date, no other studies have investigated the effects of GCS or PDT on TOS and OSI values in patients with OLP or similar disorders, highlighting the need for further research in this area. These findings suggest that both modalities effectively reduce the total oxidative burden in saliva.

TAC after GKS administration decreased significantly at all time points. In contrast, in the PDT group, TAC was substantially lower only immediately after treatment, whereas no significant differences compared to baseline were observed in long-term follow-up. Previous studies in this area remain inconclusive. Hashemy et al. (2016) compared serum TAC before and after a two-week course of a mouthrinse containing dexamethasone and nystatin in patients with OLP and found no significant changes. However, the short observation period, limited sample size (*n* = 25), and the use of serum rather than saliva markedly restrict comparability [36]. Conversely, Mansourian et al. (2017) reported a significant increase in salivary TAC after five weeks of triamcinolone acetonide mouthrinse. These discrepancies may arise from differences in treatment modality (rinse vs. ointment), biological material assessed, and treatment duration [37]. Notably, no studies to date have evaluated salivary TAC changes after photodynamic therapy in OLP, underscoring the innovative nature of the present analysis.

The observed effects of glucocorticosteroids on salivary redox balance can be explained by their mechanisms of action. GKS inhibit the release of pro-inflammatory cytokines and immune cell activation and reduce reactive oxygen species production [38]. Additionally, corticosteroids have been shown to suppress NF-κB expression, which is upregulated in OLP [39,40]. This results in a decrease in TOS and, due to lower ROS production, in reduced activity of antioxidant systems, explaining the observed TAC decline.

The impact of photodynamic therapy on redox status appears more complex. Activation of the photosensitizer during PDT induces the release of large amounts of ROS, which trigger apoptosis of pathological cells [41,42]. This mechanism likely accounts for the observed transient reduction in antioxidant activity immediately post-treatment. In the following months, antioxidant marker levels gradually increased, indicating restoration of redox balance. In addition, PDT has been shown to act through the upregulation of pro-inflammatory cytokines such as IL-1 and IL-6 [43]. Upon light activation, increased levels of these cytokines may lead to a short-term intensification of the inflammatory response, which promotes enhanced ROS production and facilitates the apoptosis of diseased cells. Following the elimination of pathological cells, environmental oxidative stress decreases, ultimately resulting in higher antioxidant activity levels. In the following months, antioxidant marker levels gradually increased, indicating restoration of redox balance. Moreover, Cosgarea et al. (2020) demonstrated that PDT decreases the number of CD137+ activated T lymphocytes, which attenuates chronic inflammation and, consequently, oxidative stress [44].

Comparing the two treatments, PDT demonstrated greater efficacy in restoring redox homeostasis in saliva. Immediately after therapy, TAC was significantly higher in the PDT group, and after six months it showed a trend toward higher values compared to the GKS group. Significantly lower OSI values in the PDT group immediately post-treatment further support the beneficial effect of this modality.

TOS and TAC are cumulative indicators reflecting the overall oxidative and antioxidative potential of the sample. Therefore, their determination is simpler and more cost-effective than assessing individual antioxidant enzymes, while providing comprehensive insight into redox status [14,17,18].

Importantly, our study is the first to compare the effects of photodynamic therapy and glucocorticosteroids on oxidative stress parameters in the saliva of patients with oral lichen planus, highlighting its novel character.

Limitations of the present study include the relatively short observation period. Additionally, only selected oxidative stress markers were assessed; a broader panel could have provided more in-depth insights. Another limitation is the inability to blind participants due to the distinctly different nature of the compared interventions, which raises the possibility of a placebo effect. The inclusion of a placebo control group was considered. However, it was deemed ethically unacceptable, as withholding active treatment from patients experiencing symptomatic oral lichen planus would have caused unnecessary discomfort and suffering.

In summary, the present analyses indicate that PDT more effectively and sustainably restores redox balance in saliva compared to glucocorticosteroid therapy. These findings may form the basis for further research on the standardization of photodynamic therapy protocols for OLP and confirm its advantage in reducing oxidative stress.

## 5. Conclusions

The conducted study demonstrated that both photodynamic therapy and topical corticosteroid therapy affect oxidative stress parameters in the saliva of patients with oral lichen planus. PDT proved to be a method that more effectively and durably restored redox balance compared to glucocorticosteroids.

The results constitute another step toward developing a standardized therapeutic protocol for OLP that incorporates photodynamic therapy as an effective and safe alternative to steroid-based treatment.

## 6. Patents

Szymańska et al. (2023) [24,25]. A pharmaceutical composition with mucoadhesive properties and its use. P.443813 (PL); PCT/IB2024/051420—PCT application number.

## Figures and Tables

**Table 1:** Descriptive statistics for age and gender.

Variable	N (%)
Gender	
Male	18 (20%)
Female	72 (80%)
Age	
Average age (M ± SD)	60 ± 11.7 years

**Table 2:** Salivary oxidative stress parameters measured at each time point during PDT and topical GKS.

Parameter	Measure	T0	T1	T3	T6
TOS (nmol H_2_O_2_ Equiv/min/mg protein)	Median PDT	385.1	214.3	258.9	269.9
	Mean ± SD PDT	520.2 ± 357.8	294.7 ± 199.7	363.6 ± 289.3	459.1 ± 397.0
	Min–Max PDT	124.2–1296	62.95–969.5	81.57–1489	115.8–1911
	Median GKS	454.5	236.0	241.4	250.4
	Mean ± SD GKS	572.6 ± 380.5	331.8 ± 263.8	334.7 ± 309.6	464.7 ± 498.2
	Min–Max GKS	96.34–1464	26.81–1136	25.09–1658	53.48–2674
TAC (Trolox μmol/mg protein)	Median PDT	43.17	37.36	24.37	22.5
	Mean ± SD PDT	45.07 ± 28.3	52.4 ± 69.21	38.43 ± 30.79	34.72 ± 27.52
	Min–Max PDT	9.192–114.8	3.53–485.6	3.077–122.3	8.542–103.8
	Median GKS	33.41	23.63	23.11	17.51
	Mean ± SD GKS	43.92 ± 28.19	29.07 ± 18.47	31.63 ± 22.91	28.83 ± 27.6
	Min–Max GKS	7.488–113.6	2.108–77.1	2.056–104.9	4.713–109.5
OSI	Median PDT	11.85	7.68	11.48	12.5
	Mean ± SD PDT	14.15 ± 9.197	9.578 ± 7.45	15.81 ± 16.6	19.03 ± 19.32
	Min–Max PDT	2.326–45.35	0.681–30.86	2.085–84.63	1.865–79.6
	Median GKS	12.51	11.99	11.62	15.16
	Mean ± SD GKS	18.51 ± 17.18	16.24 ± 15.26	15.29 ± 13.17	23.97 ± 39.93
	Min–Max GKS	2.101–81.42	0.855–76.04	1.778–56.64	1.313–251.9

PDT—parameter determined in PDT group; GKS—parameter determined in GKS group; T0—before treatment; T1—immediately after treatment; T3—3 months after treatment; T6—6 months after treatment; TOS—total oxidant status; TAC—total antioxidant capacity; OSI—oxidative stress index.

**Table 3:** Comparison of median values of TOS, TAC, and OSI in unstimulated saliva of patients treated with photodynamic therapy (PDT) and topical corticosteroids (GKS) at each time point.

Parameter	Time Point	PDT Median	GKS Median	*p*-Value (Mann–Whitney)
TOS (nmol H_2_O_2_ Equiv/min/mg protein)	T0	385.1	454.5	ns
	T1	214.3	236.0	ns
	T3	258.9	241.4	ns
	T6	269.9	250.4	ns
TAC (Trolox μmol/mg protein)	T0	43.17	33.41	ns
	T1	37.36	23.63	0.0304
	T3	24.37	23.11	ns
	T6	22.5	17.51	0.0941 (trend)
OSI	T0	11.85	12.51	ns
	T1	7.68	11.99	0.0248
	T3	11.48	11.62	ns
	T6	12.5	15.16	ns

TOS—total oxidant status; TAC—total antioxidant capacity; OSI—oxidative stress index; PDT—photodynamic therapy; GKS—topical corticosteroids; T0—before treatment; T1—immediately after treatment; T3—3 months after treatment; T6—6 months after treatment; ns—not significant (*p* > 0.05).

## Data Availability

Data available on request due to privacy restrictions or ethical reasons.
